# Supplementary material for: Inverse relation between structural flexibility and IgE reactivity of Cor a 1 hazelnut allergens
Source: Sci Rep. 2021 Feb 18;11:4173. doi: 10.1038/s41598-021-83705-z (PMC7892832; doi:10.1038/s41598-021-83705-z)
Supplement: Supplementary file 1 — Supplementary Information [file 41598_2021_83705_MOESM1_ESM.pdf]

Inverse relation between structural flexibility and IgE reactivity of Cor a 1 hazelnut allergens

Sebastian Führer, Anna S. Kamenik, Ricarda Zeindl, Bettina Nothegger, Florian Hofer, Norbert Reider, Klaus R. Liedl, Martin Tollinger*

* Corresponding author: martin.tollinger@uibk.ac.at

Supplementary Methods

Mass spectrometry experiments

For the determination of the accurate mass of the hazelnut Cor a 1.04 isoforms a 7 Tesla Fourier-transform ion cyclotron resonance (FT-ICR) mass spectrometer (Apex Ultra 70, Bruker Daltonics), equipped with an electrospray ionization (ESI) source was used. Unlabeled Cor a 1.0401, Cor a 1.0402, Cor a 1.0403, and Cor a 1.0404 protein samples were desalted five times with ca. 500 µL 100 mM ammonium acetate pH 6.8 and subsequently five times with ca. 500 µL H_2O. Afterwards, the samples were diluted with H_2O/CH_3OH (1:1, v/v) supplemented with 1 % acetic acid to ca. 1 µM. Data analysis was done with Bruker Compass DataAnalysis and Bruker Daltonics Bio Tools or mMass[1] (version 3.0.0).

CS-Rosetta model generation

For each isoform, 3000 structure models were generated by the program CS-Rosetta[2-5], using the NMR resonance assignments[6]. The Cα RMSD values for the 10 lowest energy structures were all below 3.5, 2.9, 3.1, and 3.3 Å for isoforms Cor a 1.0401, Cor a 1.0402, Cor a 1.0403, and Cor a 1.0404, respectively, indicating good convergence of the models. The lowest energy model for each isoform was used for initial identification and manual assignment of NOE cross-peaks.

NMR relaxation experiments

Experiments for measuring longitudinal and rotating-frame relaxation rates (R_1 and $R_{1\rho}$) and the $^{15}N\{^1H\}$ steady-state NOE (nuclear Overhauser effect)[7] for Cor a 1.0401 and Cor a 1.0404 were performed at 500 MHz. Relaxation periods for measuring R_1 rates were 11.1, 55.5, 111.0, 222.0, 333.0, 444.0, 555.0, and 666.0 ms with repeat experiments at 55.5 ms and 333.0 ms. For measuring $R_{1\rho}$ rates, relaxation periods were 10.0, 20.0, 30.0, 40.0, 50.0, 60.0, 70.0, and 90.0 ms, with repeats at 20.0 ms and 50.0 ms. For $^{15}N\{^1H\}$ steady-state NOE measurements two spectra were recorded, one with an interscan delay (d1) of 3 s and proton saturation for 1.25 s and the second experiment with d1 set to 4.25 s without proton saturation. The relaxation rates R_1 and $R_{1\rho}$ were determined from exponential fits using an in-house MATLAB fitting script. Transverse relaxation rates R_2 were derived from R_1 and $R_{1\rho}$ values as described [8], employing a 1.5 kHz spin-lock field. Order parameters S^2 were determined with the program FAST-Modelfree (version 4.15)[9], which has the model-free approach implemented[10-12].

Supplementary Figures

Supplementary Figure S1

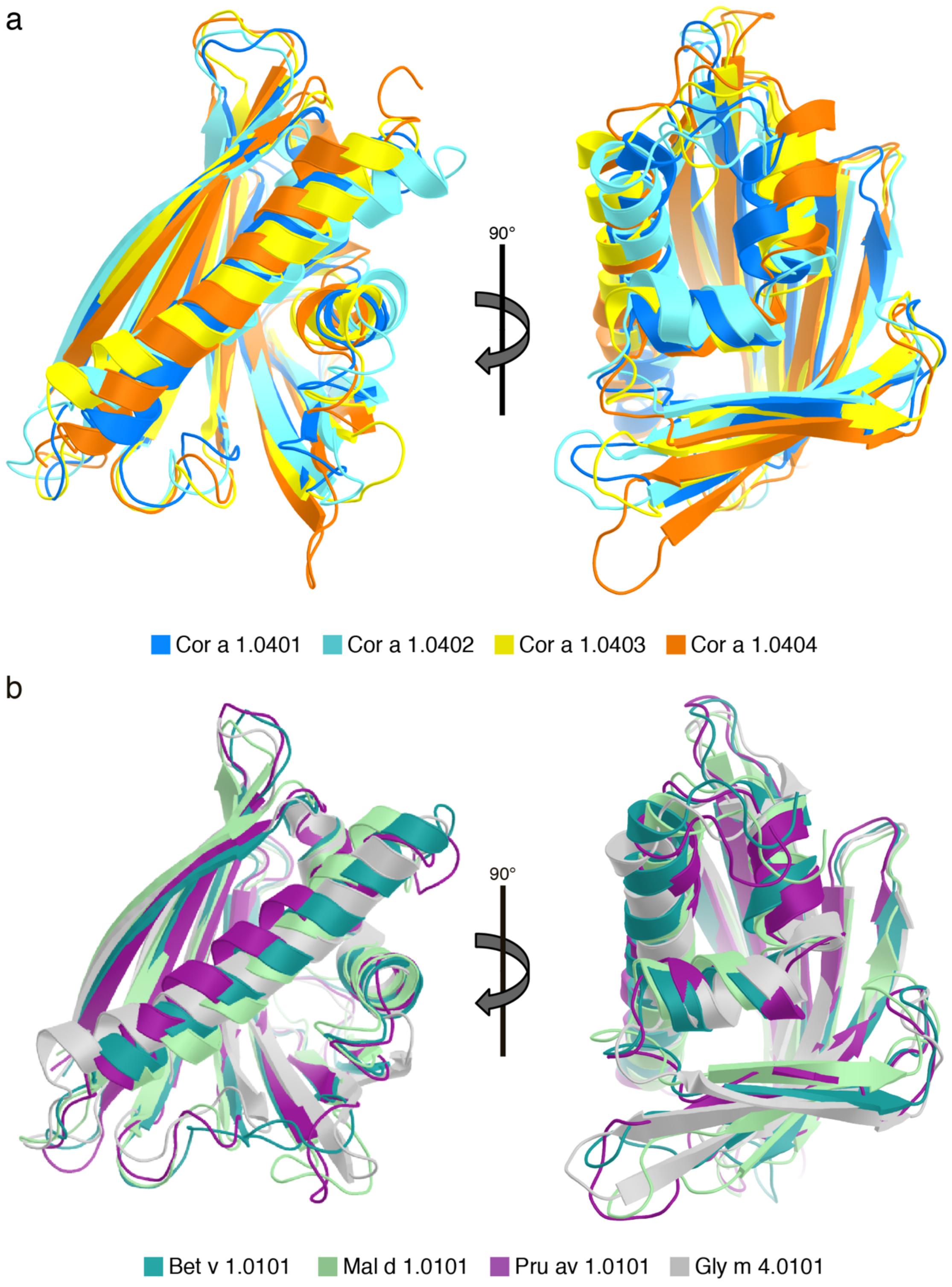

Structural comparison of the four hazelnut Cor a 1.04 isoforms with other PR-10 allergens. (a) Overlay of the solution structures of Cor a 1.0401 (blue, 6Y3H), Cor a 1.0402 (light blue, 6Y3I), Cor a 1.0403 (yellow, 6Y3K), and Cor a 1.0404 (orange, 6Y3L). (b) Overlay of the structures of Bet v 1.0101 (teal, 4A88), Mal d 1.0101 (pale green, 5MMU), Pru av 1.0101 (purple, 1E09), and Gly m 4.0101 (gray, 2K7H).

Supplementary Figure S2

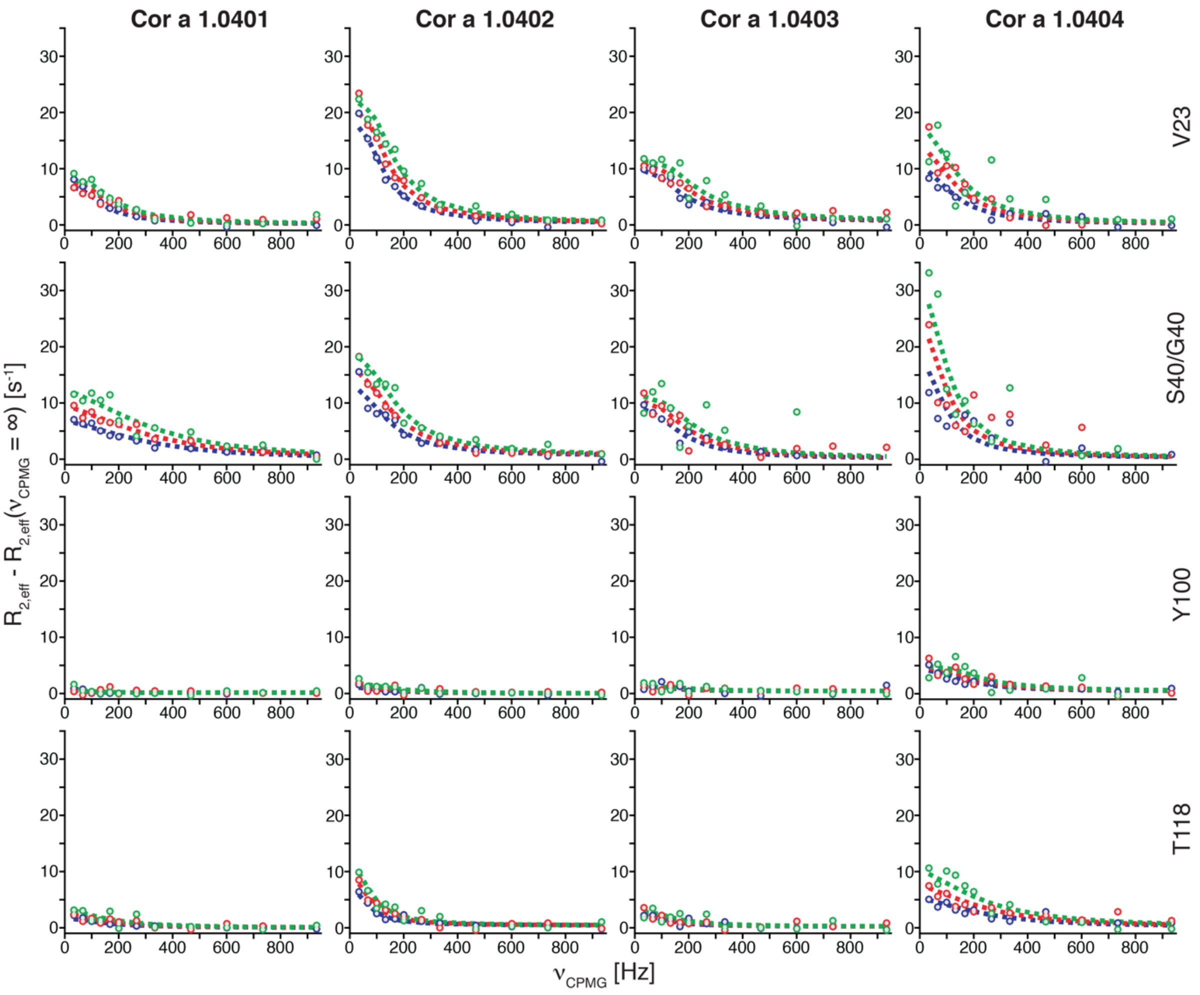

Backbone amide NH relaxation dispersion profiles of the four hazelnut Cor a 1.04 isoforms. $R_{2,eff}$ - $R_{2,eff}$ ($\nu_{CPMG} = \infty$) values at variable CPMG field strengths ν_{CPMG}, acquired at 500 MHz (blue), 600 MHz (red), and 700 MHz (green) are shown along with best-fit dashed lines. Data are shown for four representative amino acid residues in the short helix α1 (Val23), in strand β2 (Ser40 in Cor a 1.0401 and Cor a 1.0404, Gly40 in Cor a 1.0402 and Cor a 1.0403) and in the vicinity of position 99 in strands β6 (Tyr100) and β7 (Thr118). Relaxation dispersion amplitudes ($\Delta R_{2,eff}$) used in Figure 3 are given by $R_{2,eff} - R_{2,eff}(\nu_{CPMG} = \infty)$ at $\nu_{CPMG} = 0$.

Supplementary Figure S3

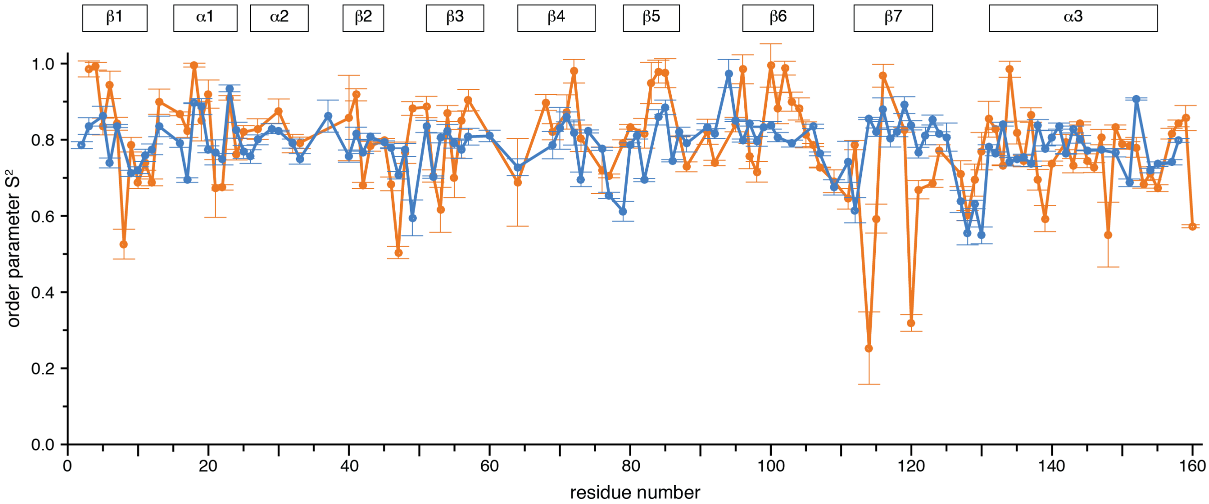

NMR-derived backbone amide NH order parameters S^2 of Cor a 1.0401 (blue) and Cor a 1.0404 (orange). Residue specific S^2 values, obtained with the program FAST-Modelfree[9], are depicted as circles and error bars are shown. Solid lines connecting residues for which experimental data are available are drawn for better visualization. Low order parameters indicate high flexibility on the picosecond to nanosecond time scale. Secondary structure elements of the hazelnut Cor a 1.04 isoforms are shown on top.

Supplementary Figure S4

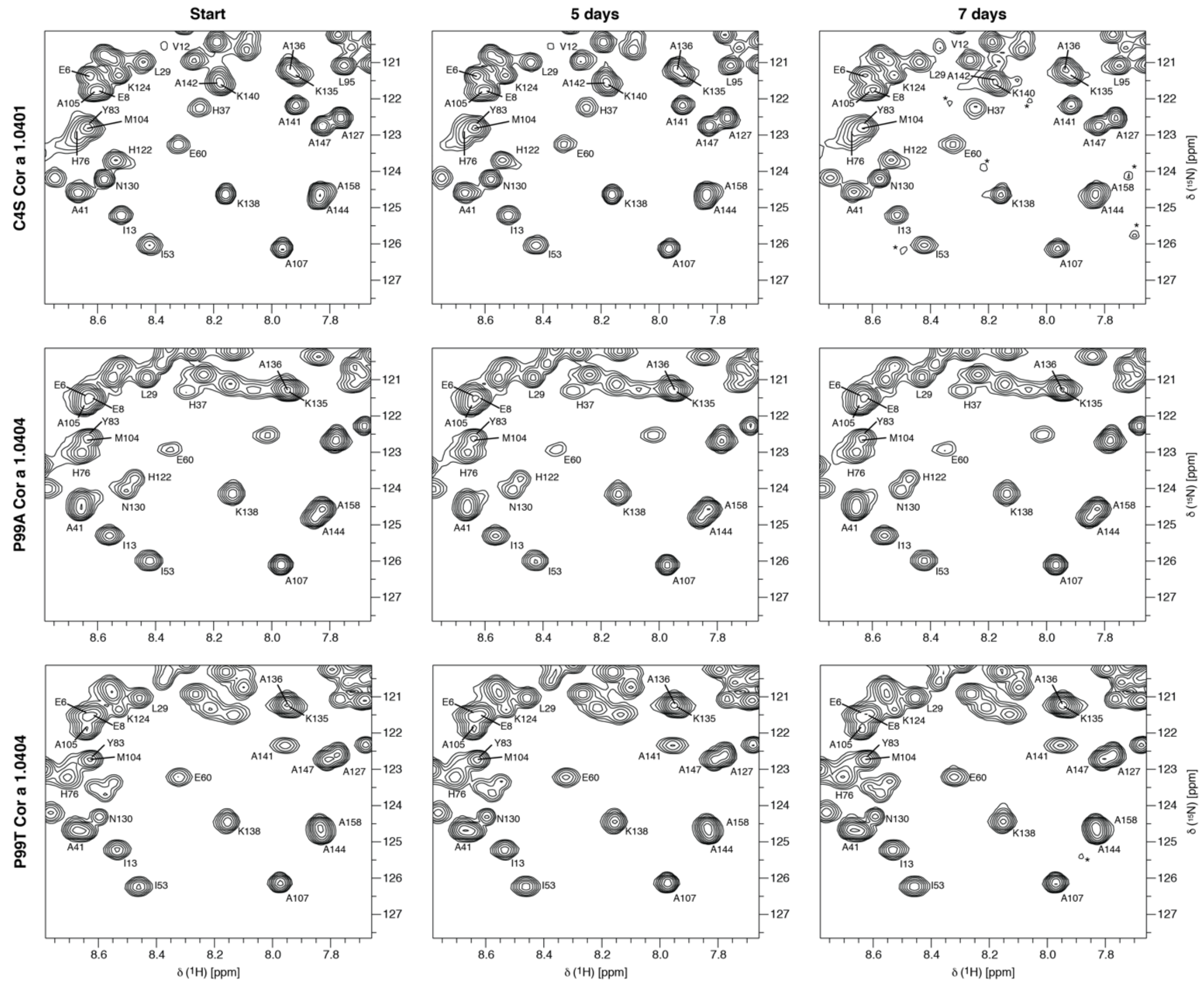

Time-dependent stability of three mutant forms of Cor a 1.0401 and Cor a 1.0404. Sections of ^{1}H-^{15}N-HSQC spectra of C4S Cor a 1.0401 (top), P99A Cor a 1.0404 (middle), and P99T Cor a 1.0404 (bottom) directly after purification, as well as five and seven days later. Tentative assignments are indicated by single letter codes and signals labeled with an asterisk indicate aggregation and degradation.

Supplementary Figure S5

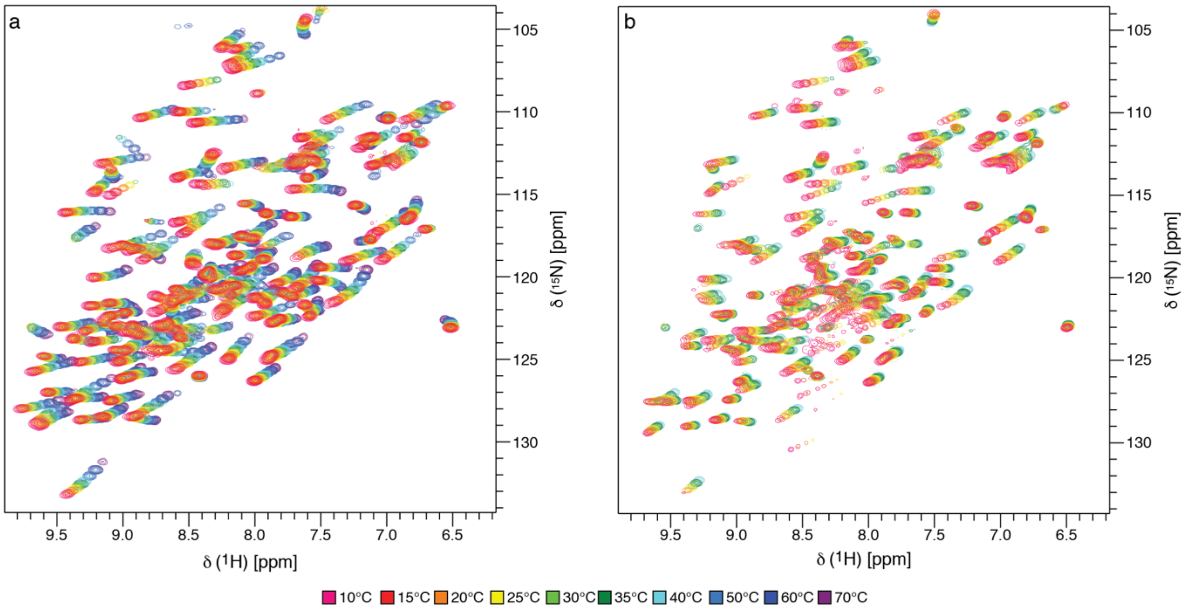

Temperature-dependent stability of Cor a 1.04 isoforms. Temperature dependent 700 MHz ^{1}H-^{15}N-HSQC spectra of Cor a 1.0401 (a) and Cor a 1.0404 (b) are superimposed (referenced to DSS). Cor a 1.0401 spectra could be acquired up to 70 °C, while Cor a 1.0404 resonances vanished above 40 °C.

Supplementary Figure S6

a

```
Cor a 1.0401         1   ATGGGCGTGTTCTGCTACGAAGATGAGGCGACCAGCGTTATCCCGCCGGCGCGTCTGTTC  60
                         ||||||||||||||*|||||||||||||||||||||||||||||||||||||||||||||
C4S Cor a 1.0401     1   ATGGGCGTGTTCTCCTACGAAGATGAGGCGACCAGCGTTATCCCGCCGGCGCGTCTGTTC  60

Cor a 1.0401        61   AAAAGCTTTGTGCTGGACGCGGATAACCTGATTCCGAAGGTTGCGCCGCAGCACTTTACC 120
                         ||||||||||||||||||||||||||||||||||||||||||||||||||||||||||||
C4S Cor a 1.0401    61   AAAAGCTTTGTGCTGGACGCGGATAACCTGATTCCGAAGGTTGCGCCGCAGCACTTTACC 120
```

b

```
C4S Cor a 1.0401   241 AAGTACTGCTATAGCATCATTGAAGGTGGCCCGCTGGGTCACACCCTGGAAAAAATCAGC 300
                       |||||||||||||||||||||||||||||||||||||||||||||||||||||||||**|
P99A Cor a 1.0404  241 AAGTACTGCTATAGCATCATTGAAGGTGGCCCGCTGGGTCACACCCTGGAAAAAATCGCC 300

C4S Cor a 1.0401   301 TACGAGATTAAAATGGCGGCGGCGCCGCACGGTGGCGGTAGCATCCTGAAGATTACCAGC 360
                       ||||||||||||||||||||||||||||||||||||||||||||||||||||||||||||
P99A Cor a 1.0404  301 TACGAGATTAAAATGGCGGCGGCGCCGCACGGTGGCGGTAGCATCCTGAAGATTACCAGC 360
```

c

```
C4S Cor a 1.0401   241 AAGTACTGCTATAGCATCATTGAAGGTGGCCCGCTGGGTCACACCCTGGAAAAAATCAGC 300
                       ||||||||||||||||||||||||||||||||||||||||||||||||||||||||||*|
P99T Cor a 1.0404  241 AAGTACTGCTATAGCATCATTGAAGGTGGCCCGCTGGGTCACACCCTGGAAAAAATCACC 300

C4S Cor a 1.0401   301 TACGAGATTAAAATGGCGGCGGCGCCGCACGGTGGCGGTAGCATCCTGAAGATTACCAGC 360
                       ||||||||||||||||||||||||||||||||||||||||||||||||||||||||||||
P99T Cor a 1.0404  301 TACGAGATTAAAATGGCGGCGGCGCCGCACGGTGGCGGTAGCATCCTGAAGATTACCAGC 360
```

Coding sequence alignment of the Cor a 1.04 mutants (a) C4S Cor a 1.0401, (b) P99A Cor a 1.0404, and (c) P99T Cor a 1.0404. The upper line indicates the sequence of the template and the lower line the sequence of the site-directed mutagenesis product. Lines represent concordant nucleotides, whereas different nucleotides are highlighted by asterisks. Successful mutations (codons) are highlighted by red boxes. Sequencing was performed by Microsynth AG (Balgach, Switzerland).

References

1 Strohalm, M., Kavan, D., Novak, P., Volny, M. & Havlicek, V. mMass 3: a cross-platform software environment for precise analysis of mass spectrometric data. *Anal Chem* **82**, 4648-4651, doi:10.1021/ac100818g (2010).

2 Bax, A., Ikura, M., Kay, L. E., Torchia, D. A. & Tschudin, R. Comparison of different modes of two-dimensional revers-correlation NMR for the study of proteins. *J Magn Reson* **86**, 304-318, doi:10.1016/022-2364(90)90262-8 (1990).

3 Shen, Y. *et al.* De novo structure generation using chemical shifts for proteins with high-sequence identity but different folds. *Protein Sci* **19**, 349-356, doi:10.1002/pro.303 (2010).

4 Shen, Y. *et al.* Consistent blind protein structure generation from NMR chemical shift data. *Proc Natl Acad Sci U S A* **105**, 4685-4690, doi:10.1073/pnas.0800256105 (2008).

5 Shen, Y., Vernon, R., Baker, D. & Bax, A. De novo protein structure generation from incomplete chemical shift assignments. *J Biomol NMR* **43**, 63-78, doi:10.1007/s10858-008-9288-5 (2009).

6 Führer, S., Zeindl, R. & Tollinger, M. NMR resonance assignments of the four isoforms of the hazelnut allergen Cor a 1.04. *Biomol NMR Assign* **14**, 45-49, doi:10.1007/s12104-019-09918-6 (2020).

7 Mittermaier, A. & Kay, L. E. New tools provide new insights in NMR studies of protein dynamics. *Science* **312**, 224-228, doi:10.1126/science.1124964 (2006).

8 Trott, O. & Palmer, A. G., 3rd. R1rho relaxation outside of the fast-exchange limit. *J Magn Reson* **154**, 157-160, doi:10.1006/jmre.2001.2466 (2002).

9 Cole, R. & Loria, J. P. FAST-Modelfree: a program for rapid automated analysis of solution NMR spin-relaxation data. *J Biomol NMR* **26**, 203-213, doi:10.1023/a:1023808801134 (2003).

10 Clore, G. M. *et al.* Deviations from the simple two-parameter model-free approach to the interpretation of nitrogen-15 nuclear magnetic relaxation of proteins. *Journal of the American Chemical Society* **112**, 4989-4991, doi:10.1021/ja00168a070 (1990).

11 Lipari, G. & Szabo, A. Model-free apporach to the interpretation of nuclear magnetic resonance relaxation in macromolecules. 1. Theory and range of validity. *Journal of the American Chemical Society* **104**, 4546-4559, doi:10.0121/ja00381a009 (1982).

12 Lipari, G. & Szabo, A. Model-free approach to the interpretation of nucelar magnetic resonance relaxation in macromolecules. 2. Analysis of experimental results. *Journal of the American Chemical Society* **104**, 4559-4570, doi:10.021/ja00381a010 (1982).
